# Patterns and determinants of breastfeeding and complementary feeding practices of Emirati Mothers in the United Arab Emirates

**DOI:** 10.1186/1471-2458-13-171

**Published:** 2013-02-25

**Authors:** Hadia Radwan

**Affiliations:** 1 Sr. Nutritionist in Community Nutrition Department, Tawam Hospital, Al Ain, Abu Dhabi, United Arab Emirates; 2 Community Nutrition Department, Tawam Hospital, P.O. Box 17831, Al Ain, United Arab Emirates

**Keywords:** Breastfeeding, Exclusive breastfeeding, Complementary feeding, Supplementation, United Arab Emirates

## Abstract

**Background:**

Breastfeeding is the preferred method of feeding for the infant. The present study aimed at investigating the different infant feeding practices and the influencing factors in the United Arab Emirates (UAE).

**Methods:**

A convenient sample of 593 Emirati mothers who had infants up to 2 years of age was interviewed. The interviews included a detailed questionnaire and conducted in the Maternal and Child Health Centers (MCH) and Primary Health Centers (PHC) in three cities.

**Results:**

Almost all the mothers in the study had initiated breastfeeding (98%). The mean duration of breastfeeding was 8.6 months. The initiation and duration of breastfeeding rates were influenced by mother’s age (P<0.034)and education(P<0.01), parity(OR=2.13; P<0.001), rooming in(OR=21.70; P<0.001), nipple problem(P<0.010) and use of contraception(P<0.034). As for the feeding patterns, the results of the multiple logistic analyses revealed that rooming in (OR=4.48; P<0.001), feeding on demand (OR=2.29; P<0.005) and feeding more frequently at night (P<0.001) emerged as significant factors associated with exclusive or predominantly breastfeeding practices. Among the 593 infants in the study, 24.1% had complementary feeding, 25% of the infants were exclusively breastfed, and 49.4% were predominantly breastfed since birth. About 30% of the infants were given nonmilk fluids such as: Anis seed drink (Yansun), grippe water and tea before 3 months of age. The majority of the infants (83.5%) in the three areas received solid food before the age of 6 months. A variety of reasons were reported as perceived by mothers for terminating breastfeeding. The most common reasons were: new pregnancy (32.5%), insufficient milk supply (24.4%) and infant weaned itself (24.4%).

**Conclusions:**

In conclusion, infant and young child feeding practices in this study were suboptimal. There is a need for a national community-based breastfeeding intervention programme and for the promotion of exclusive breastfeeding as part of a primary public health strategy to decrease health risks and problems in the UAE.

## Background

The first two years of life are critical stages for a child's growth and development. Exclusive breastfeeding for six months and continued breastfeeding with safe, appropriate and adequate feeding is recommended as a global health policy in both developing and developed countries [1]. Healthcare standards are considered to be generally high in the United Arab Emirates due to increased government spending during strong economic years. Improved standards of living and health services have led to changes in morbidity and mortality rates. The infant mortality rate declined significantly in the UAE. Mortality rate for infants under five in the UAE decreased from 17 per 1000 live birth in 1990 to 8 per 1000 in 2008 [2]. Infectious diseases have gradually declined, and chronic non-communicable diseases (NCD’s) are being more prevalent.

It was noted that nutrition-related chronic diseases have increased dramatically in recent years and within a very short period of time; they are the major causes of morbidity and mortality in the UAE (Ministry of Health Report, 2009). Musaiger [3] reported that in addition to changes in lifestyle and physical activities in Middle Eastern countries, the decline in exclusive breastfeeding and high dependence on bottle-feeding are also important factors associated with the increase in the prevalence of obesity. The World Health Organization (WHO) [4] reported that promotion of breastfeeding may contribute to the prevention of childhood obesity. Since breastfeeding represents one of the earliest nutritional experiences of newborns, the first few months after birth may be a critical window for the development of obesity later on in life [5]. In the past few years, several systematic reviews indicated that increases in diseases such as diabetes, obesity and CVD are likely to be caused by a decrease in the practice of breastfeeding [6-8]. The same studies also revealed that these chronic diseases extend beyond infancy and affect the overall health of a nation. This would suggest that obesity prevention begins with breastfeeding.

It should be noted that in Middle Eastern countries, especially in Islamic societies, breastfeeding has a religious basis. Mothers continue to nurse their children for at least two years [9]. However, modernization as well as the aggressive promotion and marketing campaigns of infant formula and other baby food have influenced the traditions of breastfeeding. This resulted in the increased use of bottle-feeding, and mothers ignored the Quranic recommendations of breastfeeding for two years [10,11]. In the UAE, limited research reported suboptimal infant feeding practices. Mixed feeding was the norm, and many mothers introduced solid food, liquid food or formula to their infants as early as one month about 50% of the mothers stopped breastfeeding before their infants reached the age of three months, and 40% did not even attempt to breastfeed [12]. In 1992, the National Nutrition Survey in the UAE [13] showed that 42% of mothers gave their children the bottle during the first month, 21% during the second and 9% during the third month. Al Mazroui et al. [14] reported that 70% of infants received non-milk supplements during the first month. In another study in Al Ain, 76.1% of infants were given milk supplements before the end of their first month, and only 28% of the mothers exclusively breastfed their infant [15]. In a recent study in the UAE, the exclusive breastfeeding rate was 76.5% on day one, 48.4% at one month and 13.3% at six months [16]. It is worth mentioning that the UAE is a multiethnic, multicultural community. As a consequence, the results of these studies varied and reflected the different breastfeeding practices of all residents, who were from different nationalities, ethnic groups and cultures. These studies did not give an accurate depiction of the breastfeeding practices of local Emirati mothers, about whom the policy makers in the UAE are mostly concerned.

The aim of this study is to evaluate breastfeeding and complementary feeding practices of Emirati mothers in the UAE, and to compare them to the guidelines and recommendations of the World Health Organization.

## Methods

This study was conducted in the MCH (Maternal and Child Health) and Primary Health Care centers (PHC) centers in Abu Dhabi, Dubai and Al Ain. These centers are homogenous as they are governmental clinics that render maternal and child health services for Emirati mothers for free. Almost all Emirati mothers with children less than five years old visit these centers for the vaccination program for their infants. A multi-stage cluster sampling technique was used to collect data. In the first stage cluster sampling, a list of the MCH centers within each city was obtained and enumerated with four out of ten centers from each city being selected using simple random sampling. Then a convenient sample of 200 subjects was selected from the four centers from each city with sizes proportionate to sizes of the clinic. Sampling was based on the number of eligible mothers visiting the MCH centers. The researcher visited the MCH centers a fixed number of times at different times and days and recruited all eligible mothers. Mothers meeting the inclusion criteria (i.e., Emirati (UAE nationality) with infants less than 2 years of age were interviewed in each city. None of the mothers refused to be interviewed. Each mother signed an informed consent form before they started face to face interview.

### Questionnaire

A pretested questionnaire consisting of 53 questions was used to obtain demographic information such as mother’s age, father’s and mother’s employment, maternal and paternal education level and information about the infant’s age, weight, birth order and family size. The survey also gathered information about different feeding practices, such as whether the mothers introduced prelacteal feeds and when solid and liquid supplements were introduced and the nature of these supplements. Mothers were asked how long they breastfed their infants and whether and how often they breastfeed them at night.

Excluding incomplete questionnaires from the 600 interviews, the final sample size included 593 participants.

### Definitions

Feeding patterns were categorized into four groups according to WHO and Labbok and Krasovec [17,18].

**▪ Exclusively breastfeeding:** infants only breastfed since birth; no water, formula or liquid supplements.

**▪ Predominately breastfeeding:** infants receiving breast-milk and water since birth.

**▪ Complementary feeding:** infants who were mainly breastfed, but infant formula and other liquid or non-dairy foods were included in their diet.

**▪ Bottle- feeding:** infants who received only formula milk since birth and did not breastfeed.

Other definitions included in this study:

**Duration of breastfeeding:** the infant’s age in months when the mother stopped breastfeeding.

**No. of breastfeeds at night:** how many times did the mother breastfeed her infant during the night.

**Initiation time of first breastfeed:** the time at which the mother started breastfeeding after delivery. **Early breastfeeding** is considered the first feed during the first hour.

**Rooming in**: placing the infant with the mother after delivery.

Education level was categorized according to school education level:

**Illiterate:** cannot read or write.

**Primary:** read and write and have completed elementary education only.

**High school:** have completed secondary education.

**Higher education:** have completed higher education than high school.

### Data analysis

The data were analyzed using the Statistical package for Social Sciences (SPSS) version 17 (SPSS Corporation, Chicago, IL). Percentage distributions of sample characteristics were computed to describe the study participants. Comparative statistics were calculated using the Chi square (χ2) test for categorical variables and one-way analysis of variance (ANOVA) for continuous variables as preliminary test. Binary Logistic regression analysis was used to examine the relationship between the initiation time of first breastfeed (≤1 hr =1 or >1 hr=0), and feeding patterns (Exclusive or almost exclusive breastfeeding =1 or not =0) and a number of characteristics that were identified from the literature as having possible effects on them. The initiation time of first breastfeed is classified as either the mother had initiated breastfeeding within an hour after delivery or after that .While for feeding patterns the dependent variable is classified as either mothers exclusively or predominantly fed their infants or not. Then variables that were significant (P<0.05) on bivariate analysis were analysed by multiple logistic regression analysis using forward LR selection method.

To compare the association of categorical predictors with the duration of breastfeeding, we use ANOVA whenever the normality and equal variance assumptions are satisfied. Otherwise, we use Kruskal Wallis test. Statistical significance was defined as P < 0.05

### Ethics

The Board of the Ethics Committee of the Faculty of Medicine at UAE University and the ethics committee of The University of Teesside-UK approved this study.

## Results

### Demographic characteristics

Table 1 shows the demographic characteristics of the study population. A total of 593 infants (0–2 years) of age, approximately 58% male and 42% female were included in this study. The mean infant birth weight was 3.15kg. The ages of mothers participating in this survey ranged from 17 – 45 years with a mean age of 27.9 years. More than half of the mothers in the three regions have high school education (53.5%). The majority of the mothers was multiparous (71%), housewives (84.7%), practiced rooming in (87.2%), and delivered normally (83.5%). Sixty percent of the participants did not use contraception and 88% of them breastfed their infants upon demand.

**Table 1:** Percentage distribution of the variables in the study population (n=593)

**Variables**	**No**	**%**
**Maternal Age ( years )** **		
<25	222	37.4
26 – 30	211	35.6
31 – 35	102	17.2
36+	58	9.8
Mother’s employment**		
Working	91	15.3
Not working	502	84.7
**Mother’s education level**		
Illiterate	34	5.7
Primary	73	12.0
high school	317	53.5
Higher education	169	28.5
Parity**		
Primiparous	171	28.8
Multiparous	422	71.2
**Delivery method** **		
Normal	495	83.5
C – section	98	16.5
Types of contraceptive**		
None	360	60.7
Non- Hormonal	57	29.7
hormonal	176	9.6
**Nipple problems**		
Yes	246	41.5
No	347	58.5
**Infant sex** **	345	58.2
Male	248	41.8
Female		
**Infant birth weight(kg)** **		
<2.5	88	14.8
2-3.0	182	30.7
3.1-4.0	291	49.1
>4.0	12	2.0
Rooming in**		
With the mother	517	87.2
In separate room	76	12.8
Feeding Pattern**		
No breastfeeding	10	1.7
Any Breastfeeding	583	98.3
Frequency of breast feeding^*,^**		
Upon demand	315	88.5
Scheduled	41	11.8
No. of breastfeeds at night**		
None	117	19.7
1-3	346	58.3
4-6	130	21.9
**Initiation time of first breastfeed**		
≤ one hour	470	80.6
> one hour	113	19.4

* included only infants who were still breastfeeding at the time of study.

** Chi-square analysis, P<0.005.

### Breastfeeding initiation and influencing factors

In total 98% of the mothers in this study initiated breastfeeding. Of the mothers who attempted to breastfeed in the hospital, 80.6% put their infants on their breast within one hour after delivery. While the rest delayed breastfeeding their babies till after one hour postpartum. About three quarters(73%) of the mothers stated that they did not give their infants any fluids(even water) except breast milk during their stay at the hospital, while the rest of the infants received supplemental fluids with breast milk such as formula milk, water, hospital fluids, crushed dates and yansun drink.

The bivariate analysis of the factors associated with the initiation time of the first breastfeed after delivery is shown in Table 2. It was noted that multiparous mothers were most likely to initiate breastfeeding within an hour than primiparous mothers. Moreover, working mothers were most likely to delay initiation time of the first breastfeeding till after an hour as compared to non working mothers. Mothers who had delivered cesarean section were significantly less likely to initiate first breastfeed within an hour than mothers who had normal delivery. Also infants who were kept with their mothers in the same room after delivery were significantly more likely to be breastfed earlier than infants who were kept in separate room. Infants who had normal birth weight (2.5-4.0 kg) were most likely to be breastfed earlier than infants with low birth weight (<2.5 kg).

**Table 2:** **Binary logistic regression for the predictors associated with the initiation time of the first breastfeed (n=587)**^**1**^

**Variables**	**No.**	OR*	95% CI**	**P value**
**Infant Sex**				**0.898**
Male	340	1		
Female	247	1.23	0.67-1.51	
**Mother’s age**				**0.291**
<25	218	1		
25-30	210	0.98	0.61-1.55	
30-35	102	0.64	0.37-1.09	
>35	57	0.68	0.35-1.33	
**Parity**				**0.000**
Primiparous	168	1		
Multiparous	419	2.26	1.51-3.39	0.000
**Mother’s Occupation**				**0.011**
Working	91	1		
Not working	496	1.090	1.17-3.08	0.009
**Infant birth weight(kg)**				**0.001**
<2.5kg	86	1		
2.5-3.0	181	1.93	1.11-3.36	0.019
3.1-4.0	289	2.90	1.71-4.93	0.000
>4.0	12	0.87	0.25-2.97	0.826
**Mother’s level of education**				**0.248**
Illiterate	33	2.17	0.79-5.96	
primary	72	1.16	0.62-2.19	
high school	314	1.43	0.93-2.20	
Higher education	168	1		
**Rooming in**				**0.000**
With mother	517	17.64	9.63-32.32	0.000
In separate room	70	1		
**Delivery Method**				**0.001**
Normal	491	2.188	1.36-3.49	0.001
C- Section	96	1		

*odd ratio ** Confidence interval.

^1^ six out of 593 mothers did not attempt to breastfeed their infants.

### Breastfeeding duration and influencing factors

Table 3 shows a number of factors that were associated with breastfeeding duration in this study. It is noted that the mean duration of breastfeeding was 8.6±5.8 months. Multiparous Mothers breastfed their infants for a significantly longer period (9.5±6.8 months) than did primiparous mothers (7.6±5.0 months) (P<0.024). As the mother’s age increased, the duration of breastfeeding increased as well: from 7.2±5.1 months for mothers less than 25 years old to 10.0±6.3 months for mothers older than 35 years of age. Mother’s education level was significantly related to the breastfeeding duration (P<0.001); mothers with primary education breastfed for longer periods (13.1±6.2 months) than did mothers of other educational levels. Also, mothers who did not have nipple problems breastfed for significantly longer periods (9.7±6.7 months) than did mothers who suffered from nipple problems (7.5±5.4 months). Moreover, mothers who either did not use any contraception or used non-hormonal contraception breastfed their infants for a significantly longer period (9.0-10.0 months) than did mothers who used hormonal contraception (7.1±5.8 months) (P<0.025). In addition, mothers who used to breastfeed more frequently during the night (4–6 times) breastfed their infants significantly longer than did mothers who did not breastfeed their infants during the night (11.7±6.4 months vs. 6.1±5.0 months respectively) (P<0.001).

**Table 3:** Factors associated with the duration of breastfeeding * (n=234^**1**^**)**

**Factors**	**No.**	**Duration of breastfeeding Months (mean± SD)**	**P value**
**Infant sex**			0.783
Boy	144	8.7 ± 6.1	
Girl	90	8.4 ± 5.6	
**Parity**			0.024
Primiparous	86	7.6 ± 5.0	
Multiparous	148	9.5 ± 6.8	
**Mother’s age**			0.034
<25	88	7.2 ± 5.1	
25-30	77	9.7 ± 7.0	
31-35	42	9.5 ± 6.5	
>35	27	10.0 ± 6.3	
**Mother’s educational level**			0.001
Illiterate	15	9.5 ± 8.3	
primary	24	13.1± 6.2	
High school	124	7.6 ± 5.6	
Higher Education	70	9.2 ± 6.0	
**Mother’s occupation**			0.261
Working	34	8.9± 6.6	
Not working	200	8.8± 6.2	
**Rooming in**		0.903	
With the mother	200	8.7± 6.3	
In Separate room	34	8.9± 5.9	
**Frequency of breastfeeding**			0.157
Upon demand	200	8.4± 5.3	
Schedule	34	7.6± 5.1	
**Nipple problems**			0.010
Yes	95	7.5± 5.4	
No	139	9.7± 6.7	
**Use of contraceptive**			0.025
None	138	9.4± 6.1	
Non Hormonal	28	10.0±7.3	
Hormonal	68	7.1± 5.8	
**No. of breastfeeds at night**			0.001
None	87	6.1± 5.0	
1-3	96	9.7 ± 6.2	
4-6	51	11.7 ± 6.4	

* ANOVA is used whenever the normality and equal variance assumptions are satisfied. Otherwise, we use Kruskal Wallis test.

^1^ included only infants who were breastfed and stopped breastfeeding at the time of the study.

### Infant feeding patterns

Infants in the study were categorized, according to their feeding patterns since birth, into four categories: exclusive breastfeeding, predominant breastfeeding, complementary feeding and formula feeding.

Among the 593 infants in this study, 24.1% had complimentary feeding since birth, and only 10 mothers exclusively bottle-fed their infants (1.7%). About a quarter of the infants were exclusively breastfeeding and about half of them were predominantly breastfed since birth. The percentage of infants who were either exclusively or predominantly breastfed for 4 or 6 month was calculated, the results showed that 7.4% (n=44) of the infants were exclusively breastfed for 4 months while only 1.9% (n=11) breastfed exclusively for 6 months. Moreover, the percentage of infants who were predominantly breastfed for 4 months was 18.0% (n=106), and the percentage of infants who were predominantly breastfed for 6 months was 7.1% (n=42).

### Factors influencing breastfeeding practices

Factors influencing exclusive and predominantly breastfeeding practices were explored using binary logistic regression analysis (Table 4).

**Table 4:** Simple binary logistic regression for the predictors associated with exclusive and predominantly breastfeeding

**Variables**	**No.**	OR*	95% CI**	**P value**
**Infant Sex**				0.058
Male	345	1		
Female	248	0.69	0.47-1.01	
**Mother’s age**				0.099
**<**25	222	1		
26-30	211	0.91	0.58-0.43	
30-35	102	1.66	0.99-2.78	
>35	58	1.43	0.76-2.71	
**Mother’s level of education**				0.010
Illiterate	34	2.12	0.87-5.16	
Primary	73	2.12	1.11-4.07	
High school	317	1.90	1.26 -2.87	
Higher education	169	1		
**Mother’s Occupation**				0.009
Working	90	1		
Not Working	493	1.89	1.17-3.07	
**No. of breastfeeds at night**				0.006
None	117	1		
1-3	346	1.97	1.25 -3.09	
4-6	130	2.19	1.25 -3.84	
**Parity**				0.002
Primiparous	171	1		
Multiparous	422	1.83	1.24 -2.71	
**Rooming in**				0.000
with mother	517	5.87	3.54 -9.75	
In separate room	76	1		
**Frequency**				0.006
Scheduled	43	1		
Upon demand	320	2.76	1.37 -5.52	
**Nipple problems**				0.072
Yes	246	1		
No	347	1.41	0.96 -2.07	
**Type of Delivery**				0.001
Cesarean	98	1		
Normal	495	2.20	1.40 - 3.48	
**Use of contraception**				0.262
None	360	1		
Non-Hormonal	176	1.18	0.58-1.59	
Hormonal	57	0.83	0.43-1.59	

*odds ratio ** Confidence interval.

The analysis showed that mother’s education level, occupation, parity, rooming in, type of delivery, frequency of breastfeeding and the numbers of breastfeeds at night were significant factors associated with exclusive and predominant breastfeeding patterns. Housewives were two times more likely to provide exclusive or predominantly breastfeeding of their infants than working mothers (OR= 1.89; 95% CI 1.17-3.07). Moreover, multiparous mothers were 1.8 times more likely to either exclusively or predominantly breastfeed their infants than primiparous mothers (OR =1.83; 95% CI 1.24-2.71). The odds of either exclusive or predominantly breastfeeding the infants by the mothers who completed either primary or high school educated were about two times compared to the corresponding odds for higher educated mothers. Mothers who delivered normally were more likely to either exclusively or predominantly breastfeed their infants than did those who delivered by cesarean section (OR= 2.20; 95% CI 1.40-3.48). Infants who were breastfed upon demand (OR= 2.76; 95% CI 1.37-5.52) were more likely to be either exclusively or predominantly breastfed than did infants who were breastfed on schedule. Moreover, the odds for mothers who kept their infants with them in the same room after delivery (rooming in) to either exclusively or predominantly breastfeed their infants were six times the corresponding odds for the mothers who kept their infants in separate rooms (OR= 5.87; 95% CI 3.54-9.75). Also, the odds for mothers who breastfed their infants at night to either exclusively or predominantly breastfeed their infants were double the corresponding odds for the mothers who did not breastfeed their infants during the night (P<0.001).

### Multiple logistic regression analysis of the variables influencing the infant feeding practices

In order to identify the most predictive factors in infant feeding practices in this study, only significant variables that were associated with the infant feeding practices in the simple binary logistic test were analysed further using multiple logistic regression models. Rooming in (OR=4.48; 95% CI 2.14-9.39; P<0.001), breastfeeding on demand (OR=2.92; 95% CI 1.39-6.14; P<0.005), and breastfeeding at night for 1–3 (OR=3.37; 95% CI 1.27-8.96; P<0.015) or for 4–6 times each night (OR=3.50; 95% CI 1.16-10.53; P<0.026) were the factors that remained in the equation as associated with infant’s exclusive or predominantly breastfeeding, allowing for intercorrelation.

### Introduction of liquids and solid supplementation

#### Non-milk liquid supplementation

Mothers were also asked if they gave their infants any traditional drinks or fluid supplements while breastfeeding.

About 70% of the mothers said that they did not give any fluid supplements to their infants below 3 months of age. However, 15.3% of the respondents said that they had given grippe water, 10.1% had given yansun and 2.5% had given tea to their infants during the first three months of the infant’s age.

#### Milk and solid supplementation

It is generally recommended by the WHO that solids be introduced to the infants at 6 months of age, as infants are physiologically and developmentally ready for new foods, textures and modes of feeding at this age.

**Figure 1:**
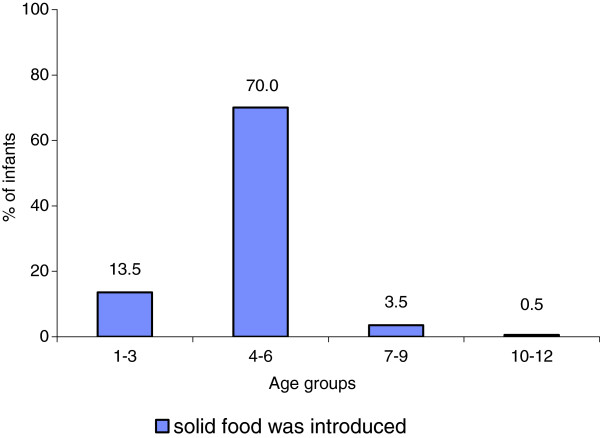
Percentage of infants receiving solid foods for the first time in different age groups.

The majority of the infants in this study were introduced to solid foods before the recommended age of 6 months (83.5%). About 13.5% of them were given solids, mostly home-made cereals or ready-made cereals, before the age of 3 months (Figure 1).

Mothers were asked to recall the type of foods introduced to their infants as well as the age at which these foods were offered to the infant. Table 5 lists the foods that were introduced to the infants and the mean infant age.

**Table 5:** Mean age at which each of the complementary food was added

**Complementary food**	**Mean age ± SD**	**Median**	**Min**	**Max**
Formula milk	3.8± 3.8	3.00	1	24
Home made cereal	4.9 ± 1.7	4.00	1	13
Ready-made cereal	4.6 ± 1.1	4.00	2	12
Fruits	5.1 ± 1.6	5.00	2	13
Vegetables	5.9 ± 2.5	5.00	3	24
Legumes	7.8 ± 2.6	7.00	3	18
Eggs	7.4 ± 2.1	7.00	3	18
Meat	8.8 ± 3.1	8.00	3	24
Chicken	8.6 ± 2.9	8.00	3	24
Fish	9.1 ± 3.3	8.00	4	24

It was noted that the mean infant age (±SD) at which formula milk was introduced was 3.8 ± 3.8 months. Home made cereals and ready made cereals were introduced to infants at 4.9 ± 1.7months, and 4.6 ± 1.1 months respectively. Fruits and vegetables were introduced at a mean infant age (±SD) of 5.1± 1.6 months, and 5.9 ± 2.5 months respectively. Legumes and eggs were given to the infants at about 7 months, while meat and chicken were introduced at the age of about 8 months. Fish was introduced later on average at 9 months.

#### Reasons for termination of breastfeeding

Mothers in this study were asked why they stopped breastfeeding. The main reasons for stopping breastfeeding as stated by the mothers were mainly new pregnancy and breastmilk insufficiency. Some mothers said that they terminated breastfeeding because they got pregnant again (32.9%; n=77), while about 24.4% (n=57) of the mothers stopped breastfeeding because they said they did not have sufficient breast milk. Others said that the child refused to continue breastfeeding (24.4%; n=57). This reason could also be related to breastmilk insufficiency because may be the child refused to continue breastfeeding because there was not sufficient breastmilk to satisfy his hunger. Other reasons for terminating breastfeeding were child’s reaching weaning age (6.0%; n=14) and mother’s sickness (5.6%; n=13). Mother’s refusal, infant sickness and mother’s returning to work were reported by 6.8% (n=16) of the interviewees as the reasons behind stopping breastfeeding.

Mothers in this study followed different methods for terminating breastfeeding. Half of them (n=86, 50%) physically distanced themselves from their infants. Some even said that they had sent their children to relatives, most often the children’s grandparents, for several days. Others (n=43, 25%) started introducing formula milk so their children would get used to it. While some of the mothers (n=43, 25%) rubbed their nipples with lipstick or a bitter substance called “sabr” so that the infant would dislike breastfeeding.

## Discussion and conclusion

The UAE have embraced the WHO recommendations, and the Ministry of Health issued an infant feeding policy which recommends six months of exclusive breastfeeding. However, translation of these policies into action in the UAE would require immense planning and strong implementation programmes and strategies. Despite many efforts and plans to promote breastfeeding in the UAE, there are no clear national targets, strategies or action plans to protect, promote and support appropriate feeding practices of infants and young children. The UAE should plan an infant feeding policy in the context of WHO/UNICEF Global Strategy for Infant and Young Child Feeding (IYCF) [19]. IYCF reported that once a strategy and an action plan are put in place, then countries will be able to proceed steadily and measure their progress towards established targets.

There are several limitations that need to be considered when interpreting the results of this study. One important limitation of this study is recall bias due to the retrospective nature of the data collection, possibly resulting in over or under estimation of actual breastfeeding practices. Although recall biases cannot be avoided, the author conducted all interviews to ensure consistent technique and interpretation of the answers. It should be also noted that epidemiological studies of this kind do not establish causality but may suggest associations.

Another limitation of this study is the representation of all the Emirati women who had babies and breastfeeding during the period of research. The generalisability of the results is limited by the element of convenience sampling – samples were drawn at the MCH and PHC clinics, which increase the likelihood of some types of people being selected rather than others. However, (a) strong attempts were made to match the demographic characteristics of the general population of mothers, and (b) a large proportion (more than 90%) of Emirati mothers do use the clinics for following up with the vaccination programs of their infants, as required by the Ministry of Health.

The findings in this study showed that initiation time of the first breastfeed of the whole population studied was high (98%). Although this high initiation rate is encouraging compared to Western countries, the premature introduction of supplementary feeds, the early cessation of breastfeeding and failure to exclusively breastfeed are great concerns. Despite the efforts of the Ministry of Health and the Health Education programs on breastfeeding, many participants still introduced either prelacteal fluids or supplementary food before the infant reached six months of age.

A number of socio-demographic variables were associated with the initiation and duration of breastfeeding in this study. In general, multiparous mothers were likely to initiate and exclusively breastfeed for longer periods than primiparous mothers. The association of parity with breastfeeding initiation, exclusivity and duration was investigated in several studies and similar results were reported [20-22]. However, education as a predictor differs between developing and developed countries. Educated mothers in most developed countries have returned to breastfeeding [23,24] while in developing countries, mothers with high education have increasingly switched to bottle feeding or mixed feeding [25,26].This finding has been explained by Abada et al. [27] that higher education in developing countries is associated with the adoption of modern ideas, often leading to the abandonment of traditional practices such as breastfeeding.

Although the majority of Emirati mothers, in this study, breastfed their infants, only 25% of them were exclusively breastfed for six months. Similarly, low levels of exclusive breastfeeding were recorded in other countries [28-30]. Higher rates of exclusive breastfeeding have been reported in New Zealand [31] and Norway [32], where 42% of the infants were exclusively breastfed for 4 months. However, even in these countries, exclusive breastfeeding declined to 7% at six months of age. This means that there still exists a need for encouraging mothers to continue exclusive breastfeeding till the infants are 6 months old.

Providing children with water as early as one month of the infant’s age, is a normal practice in UAE as well as many other communities [33-35]. Emirati mothers justified this practice by stating that the UAE have a hot climate, and infants need to be hydrated. Early supplementation with formula milk and other liquids was also noted in this study. It should be mentioned that breast milk alone can maintain adequate water balance in young infants and supplementary fluids are not needed even in warm climates [36,37].

Caesarean delivery, contraception and nipple problems were reported in this study as influencing variables associated with breastfeeding practices. These factors have previously been shown to hinder breastfeeding and disrupt lactation [38-40].

Multiple logistic analysis of breastfeeding patterns indicated that rooming in, number of breastfeeds at night and breastfeeding on demand were the most significant predictors influencing breastfeeding patterns. Rooming in encourages demand and night feeding, and this allows frequent and close contact between the mother and the baby [41]; it also encourages the establishment of longer duration of exclusive breastfeeding [42,43].

Timely introduction of solid foods remains an important factor for healthy infant growth. The premature introduction of complementary food was of great concern in the present study. Despite the efforts of the UAE national health education programs, many participants still introduced complementary food before the baby reached six months of age. Musaiger [9] revealed that weaning foods are introduced very early in all Arabian Gulf countries. This was attributed to high purchasing power and the wide availability of commercial baby foods. Research suggested that complementary foods offered to infants before 6 months of age tend to displace breastmilk without conferring any growth advantage over exclusive breastfeeding [44]. Early introduction of other foods or drinks is an area of concern mainly because it marks the end of exclusive breastfeeding which has protective effects [45].

In the present study, the most frequently reported reason for starting weaning and terminating breastfeeding was that the mother became pregnant (32.9%). This reason was also recorded by other studies for discontinuing breastfeeding [46-48] The belief that the breast milk of pregnant women could be harmful or no longer nutritious is widely common among Gulf women [9] and women from other communities [49,50].

Other participants, in this study, stated that they stopped breastfeeding and started formula feeding because they believed that their breastmilk was insufficient and infant refused to suck. There seems to be a widespread perception of lactation insufficiency reported by other studies, and accordingly, the infant is given supplements at a very early age [42,51]. The mother’s concern about milk insufficiency could be explained by her poor understanding of the proper techniques to increase breastmilk. Contrary to this belief, most mothers are able to produce breastmilk in quantities adequate for the proper growth of their infants, even in societies where the mother’s diet is poor [38]. Daly and Hartmann [52] revealed that maternal milk production is finely tuned to the demand of the infant. Therefore, frequent and exclusive breastfeeding is critical for stimulating optimal milk production.

The results of this study suggest an urgent need to target breastfeeding education campaigns at young, primiparous, which either completed high school or higher education. Those mothers were identified by this study as the most vulnerable group who are at risk of not exclusively breastfeeding. So proper advice and breastfeeding management are required to increase awareness of exclusive breastfeeding benefits and to ensure that the problems Emirati mothers face during breastfeeding do not lead to the cessation of breastfeeding.

The promotion of breastfeeding can be a potential component of the primary public health strategies to decrease public health problems in the UAE, such as obesity and NCD’s and their related risk factors. This will contribute to improving the health, nutrition and well-being of both infants and mothers.

Moreover, the findings of this study should be the foundation of future studies that investigate the feeding patterns in the UAE, and eventually assist in creating a national infant feeding policy. There is a need for a larger more detailed study following women from before birth until they stop breast feeding their babies, the thoughts and feelings of the mother and her social networks at each stage of the process should be explored.

## Competing interests

The author declares that she has no competing interests**.**

## Authors’ contributions

HR designed the research, collected and analyzed data, and drafted the manuscript.

## Pre-publication history

The pre-publication history for this paper can be accessed here:

http://www.biomedcentral.com/1471-2458/13/171/prepub
